# Supplementary material for: Influence of temperature on developmental and biochemical traits of the red squat lobster Grimothea monodon (H. Milne Edwards, 1837) during early ontogeny
Source: PeerJ. 2025 Oct 22;13:e20278. doi: 10.7717/peerj.20278 (PMC12553365; doi:10.7717/peerj.20278)
Supplement: Supplemental Information 2 [file peerj-13-20278-s002.docx]

**Table S1:** Summary of the generalized linear model (GLM) fitted to larval stage (I–VI) and temperature (12 °C vs. 20 °C). Significant effects (p < 0.05) are shown in bold.

| *Predictors* | *Estimates* | *Std. Error* | *p* |
| --- | --- | --- | --- |
| (Intercept) | 0.68 | 0.0225447 | **<0.001** |
| Temperature | -0.03 | 0.0318831 | 0.317 |
| Larval stage [II] | 0.11 | 0.0318831 | **0.001** |
| Larval stage [III] | 0.19 | 0.0318831 | **<0.001** |
| Larval stage [IV-A] | 0.34 | 0.0318831 | **<0.001** |
| Larval stage [IV-B] | 0.52 | 0.0318831 | **<0.001** |
| Larval stage [IV-C] | 0.66 | 0.0318831 | **<0.001** |
| Larval stage [IV-D] | 0.75 | 0.0318831 | **<0.001** |
| Larval stage [IV-E] | 0.97 | 0.0318831 | **<0.001** |
| Larval stage [V] | 1.05 | 0.0318831 | **<0.001** |
| Larval stage [VI] | 1.06 | 0.0552231 | **<0.001** |
| Temperature × Larval stage [II] | -0.01 | 0.0450895 | 0.886 |
| Temperature × Larval stage [III] | -0.02 | 0.0457114 | 0.693 |
| Temperature× Larval stage [IV-A] | -0.08 | 0.0450895 | 0.073 |
| Temperature × Larval stage [IV-B] | -0.06 | 0.0450895 | 0.221 |
| Temperature × Larval stage [IV-C] | -0.04 | 0.0450895 | 0.325 |
| Temperature × Larval stage [IV-D] | 0.000999 | 0.0450895 | 0.982 |
| Temperature × Larval stage [IV-E] | -0.12 | 0.0450895 | **0.009** |
| Temperature × Larval stage [V] | -0.05 | 0.0450895 | 0.269 |
| Temperature × Larval stage [VI] | 0.08 | 0.0637661 | 0.209 |
| Observations | 191 | | |
